# Prevalence of *Clonorchis sinensis *infection in dogs and cats in subtropical southern China

**DOI:** 10.1186/1756-3305-4-180

**Published:** 2011-09-19

**Authors:** Rui-Qing Lin, Jian-Dong Tang, Dong-Hui Zhou, Hui-Qun Song, Si-Yang Huang, Jia-Xu Chen, Mu-Xin Chen, Han Zhang, Xing-Quan Zhu, Xiao-Nong Zhou

**Affiliations:** 1 State Key Laboratory of Veterinary Etiological Biology, Key Laboratory of Veterinary Parasitology of Gansu Province, Lanzhou Veterinary Research Institute, Chinese Academy of Agricultural Sciences, Lanzhou, Gansu Province 730046, PR China; 2 College of Veterinary Medicine, South China Agricultural University, Guangzhou, Guangdong Province 510642, PR China; 3 National Institute of Parasitic Diseases, Chinese Center for Disease Control and Prevention, Shanghai 200025, PR China; 4 Guangdong Institute for Animal Disease Control and Surveillance, Guangzhou, Guangdong Province 510230, PR China; 5 College of Animal Science and Technology, Yunnan Agricultural University, Kunming, Yunnan Province 650201, PR China; 6 College of Animal Science and Veterinary Medicine, Heilongjiang Bayi Agricultural University, Daqing, Heilongjiang Province 163319, PR China

## Abstract

**Background:**

Clonorchiasis, caused by *Clonorchis sinensis*, is one of the major parasitic zoonoses in China, particularly in China's southern Guangdong province where the prevalence of *C. sinensis *infection in humans is high. However, little is known of the prevalence of *C. sinensis *infection in its reservoir hosts dogs and cats. Hence, the prevalence of *C. sinensis *infection in dogs and cats was investigated in Guangdong province, China between October 2006 and March 2008.

**Results:**

A total of 503 dogs and 194 cats from 13 administrative regions in Guangdong province were examined by post-mortem examination. The worms were examined, counted, and identified to species according to existing keys and descriptions. The average prevalences of *C. sinensis *infection in dogs and cats were 20.5% and 41.8%, respectively. The infection intensities in dogs were usually light, but in cats the infection intensities were more serious. The prevalences were higher in some of the cities located in the Pearl River Delta region which is the most important endemic area in Guangdong province, but the prevalences were relatively lower in seaside cities.

**Conclusions:**

The present investigation revealed a high prevalence of *C. sinensis *infection in its reservoir hosts dogs and cats in China's subtropical Guangdong province, which provides relevant "base-line" data for conducting control strategies and measures against clonorchiasis in this region.

## Background

Clonorchiasis, caused by *Clonorchis sinensis*, is considered as one of the major parasitic zoonoses in some parts of Asia, which can cause a series of hepatic diseases such as periductal inflammation, fibrosis, pyogenic cholangitis, biliary calculi, cholecystitis, liver cirrhosis and pancreatitis, and *C. sinensis *is currently classified as carcinogenic to humans for cholangiocarcinoma [1-3]. It is estimated that about 35 million people are infected globally, and of whom approximately 15 million are in China [4,5], where human clonorchiasis is endemic in 27 provinces (including municipal cities and autonomous regions) [4-6].

Snails serve as the first intermediate hosts for *C. sinensis *and the prevalence is high (up to 15%) in some areas of China, and approximately 140 species of freshwater fishes and four species of shrimp have been recognized as the second intermediate hosts for *C. sinensis *in China [6]. Many mammals, such as cats, dogs and pigs, serve as definitive hosts or reservoir hosts for *C. sinensis *[4,7,8]. "Ingesting raw fish" is considered to be the main risk factor for human infection with *C. sinensis *[4,9,10]. Dogs and cats are the most important animal reservoirs for human infection with *C. sinensis*. There are about two hundred million dogs and one hundred million cats in China [11]. Since the 1990s, epidemiological studies of *C. sinensis *infection in dogs and cats have been conducted in many provinces, such as Guizhou, Sichuan, Guangxi, Heilongjiang, Jiangsu, and Hunan [12-18], and the prevalences are summarized in Table 1, because these data were published in local Chinese journals which are not readily accessible to international readers. In general, the prevalence of *C. sinensis *infection in dogs and cats is high but it differs in different areas. Compared with that of dogs, the prevalence of *C. sinensis *in cats is obviously higher, possibly due to their preference of eating fish.

**Table 1:** Prevalence of *Clonorchis sinensis *infection in dogs and cats in other provinces/cities of the People's Republic of China

Species	Province/city	No. tested	Positive (%)	**Test method**^**a**^	Year tested	References
Dog	Hunan	438	29.4	PE	2006-2007	Dai et al. (2009) [18]
	Heilongjiang	178	26.4	PE	1996-2004	Wang et al. (2006) [16]
	Guangxi	110	56.4	PE and FE	1990-1997	Li et al. (2002) [15]
	Sichuan	891	25.6	FE	1978-1985	Qu et al. (1997) [13]
	Jiangsu/Xinyi	99	48.5	PE and FE	1998-2006	Suo et al. (2008) [17]
	Guizhou	120	0.8	PE	1991-1993	Wang et al. (1995) [12]
Cat	Hunan/Changsha	8	62.5	PE and FE	2000	Liu et al. (2000) [14]
	Guangxi	78	64.1	PE and FE	1990-1997	Li et al. (2002) [15]
	Sichuan	201	42.3	FE	1978-1985	Qu et al. (1997) [13]
	Guizhou	36	0	PE	1986	Wang et al. (1995) [12]
	Jiangsu/Xinyi	151	62.9	PE and FE	1998-2006	Suo et al. (2008) [17]

^a ^PE: post-mortem examination; FE: faecal examination

Guangdong province is bounded by the South China Sea and is the southern gateway of China. It has the largest number of people (approximately 5.5 million) infected with *C. sinensis *because of the local custom of eating raw and undercooked freshwater fish [6]. Accompanied by the high speed development of economy and the increasing living standards during the last two decades, more and more dogs and cats are being raised and kept in increasing numbers of families as the favorable companion animals in Guangdong province. As reservoir hosts, dogs and cats play an important role in the transmission of *C. sinensis *to humans. Therefore, *C. sinensis *infection in dogs and cats not only remains a severe health problem for dogs and cats, but also poses public health concerns in China. Some surveys have shown that infection of dogs and cats with *C. sinensis *is common in other provinces of China (Table 1). However, prior to the present comprehensive survey, there had been no detailed data of *C. sinensis *prevalence in dogs and cats in Guangdong province.

In order to provide "base-line" data for the improved prevention and control of *C. sinensis *infection in dogs and cats, and for reducing the risk of human infection, the objective of the present investigation was to estimate the prevalence of *C. sinensis *infection in dogs and cats in different geographical locations in southern China's subtropical Guangdong province by post-mortem examination.

## Materials and methods

### The study site

Guangdong province is located in the southern part of mainland China, between north latitudes of 20°19' to 25°31' and east longitudes of 109°45' to 117°20', sharing borders with Hong Kong and Macao Special Administrative Regions of China. This province has a subtropical climate, with an average annual temperature of 19°C to 23°C. The province has an area of approximately 180 thousand square kilometers, and has a population of approximately 95 million. People in Guangdong have the habit of eating slices of raw freshwater fishes. Guangdong province has 13 administrative regions (cities), and dogs were sampled from each of these (Table 2). Cats were sampled from 8 of the 13 administrative regions (cities) (Table 3), which represent all the main endemic regions for *C. sinensis*.

**Table 2:** Prevalence of *Clonorchis sinensis *infection in dogs in Guangdong province, China

Cities surveyed	No. examined	No. positive	Prevalence (%)	Infection intensity (worms per dog)
Jiangmen	20	3	15	0 (17), 1-10 (1), 11-50 (1), > 100(1)
Guangzhou	14	1	7.1	0 (13), 1-10 (1)
Shantou	68	1	1.5	0 (67), 1-10 (1)
Yunfu	36	13	36.1	0 (23), 1-10 (7), 11-50 (4), 51-100 (2)
Maoming	44	0	0	0 (44)
Shaoguan	49	34	69.4	0 (15), 1-10 (12), 11-50 (9), 51-100 (5), > 100 (8)
Foshan	21	4	19.0	0 (17), 1-10 (2), 51-100 (1), > 100 (1)
Zhaoqing	42	5	11.9	0 (37), 1-10 (4), > 100 (1)
Meizhou	48	12	25	0 (36), 1-10 (3), 11-50 (2), 51-100 (4), > 100 (3)
Qingyuan	22	2	9.1	0 (20), 1-10 (2)
Shenzhen	23	9	39.1	0 (14), 1-10 (3), 11-50 (1), 51-100 (3), > 100 (2)
Yangjang	10	0	0	0 (10)
Zhanjiang	106	19	17.9	0 (87), 1-10 (13), 11-50 (4), > 100 (2)
Total	503	103	20.5	0 (400), 1-10 (49), 11-50 (21), 51-100 (15), > 100 (18)

**Table 3:** Prevalence of *Clonorchis sinensis *infection in cats in Guangdong province, China

Cities surveyed	No. examined	No. positive	Prevalence (%)	Infection intensity (worms per cat)
Jiangmen	18	12	66.7	0 (6), 1-10 (7), 11-50 (1), 51-100 (2), > 100 (2)
Guangzhou	41	30	73.2	0 (11), 1-10 (6), 11-50 (12), 51-100 (3), > 100 (9)
Shantou	47	1	2.1	0 (46), 1-10 (1)
Yunfu	20	12	60	0 (8), 1-10 (7), 11-50 (5)
Foshan	26	9	34.6	0 (17), 1-10 (4), 11-50 (3), > 100 (2)
Qingyuan	6	2	33.3	0 (4), 1-10 (2)
Yangjiang	19	9	47.4	0 (10), 1-10 (2), 11-50 (3), 51-100 (2), > 100 (2)
Zhanjiang	17	6	35.3	0 (11), 1-10 (5), 11-50 (1)
Total	194	81	41.8	0 (113), 1-10 (34), 11-50 (25), 51-100 (7), > 100 (15)

### Examination of dogs and cats for the presence of *C. sinensis *and data collection

From October 2006 to March 2008, livers from 503 adult farm dogs and 194 adult farm cats sampled randomly from the selected study regions (cities) of Guangdong province were examined for the presence of *C. sinensis *adults. These animals were treated humanely, according to the Animal Ethics Procedures and Guidelines of the People's Republic of China, and the study was approved by South China Agricultural University. Individual livers of dogs and cats were split into two lobes according to anatomical structure, then the bile ducts were opened longitudinally with a pair of scissors in 0.9% saline, and washed with the same solution until the supernatant had cleared. The sedimentary contents of bile ducts were then carefully examined with the naked eye. All worms were counted if the total number of worms was less than 100, or a proportion of all collected worms were counted if more than 100 worms were estimated to be present. Specimens were fixed in 75% ethanol after being cleared and species identification was carried out according to existing descriptions and keys (testes and vitelline glands) [4,19]. The identity of three representative recovered worms was ascertained by the amplification and subsequent sequence analysis of the internal transcribed spacers (ITS) of nuclear ribosomal DNA following methods reported previously [20].

A dog or cat was recorded as infected if at least one *C. sinensis *worm was found in that animal. The mean prevalence was calculated by dividing the number of infected animals with the total number of animals examined, and was expressed as a percentage. Infection intensity was divided into 5 grades: 0, 1-10, 11-50, 51-100, > 100 (worms per dog or cat).

## Results

The worms found in livers of dogs and cats showed classical morphological features of *C. sinensis*. The worms were flat, leaf-like, about 6-17 mm in length and 1-3 mm in width, with branched testes in tandem position and the continuously distributed vitelline glands. PCR amplification of ITS rDNA of representative worms and subsequent sequence analysis demonstrated that the examined worms had almost identical ITS sequences to that of *C. sinensis *reported previously [20] (not shown).

Of 503 examined dogs, 103 (20.5%) were positive for *C. sinensis *in Guangdong province, and the infection intensity in dogs were mainly light, with 47.6% of the positive dogs being infected with 1 to 10 adult worms in the liver, but 17.5% of the positive dogs being infected with more than 100 adult worms (Table 2). Usually, high prevalence was accompanied with high intensity. For example, the prevalence of *C. sinensis *in dogs in Shaoguan was the highest, being 69.4%, with high infection intensity--13 dogs of 34 positive dogs were infected with more than 50 worms.

Of the 194 cats, 81 (41.8%) were infected with *C. sinensis *(Table 3), and the prevalence in cats in Guangzhou, the capital of Guangdong province, was the highest, being 73.2%. The prevalence of *C. sinensis *in cats in Guangdong province was significantly higher than that in dogs (*P *< 0.05). The infection intensity was also high in cats, and even thousands of worms were found in some cats, obviously due to their eating preference for fish.

## Discussion

It is important to study the transmission and epidemiological features of *C. sinensis *infection in order to effectively control human clonorchiasis. Epidemiological studies of *C. sinensis *infection in humans and/or in intermediate hosts have been studied in South Korea, Japan, and China [2,8,10,21,22]. The prevalence of *C. sinensis *in many animal reservoir hosts such as dogs and cats have also been investigated in Korea and some provinces of China [7,8,12-18], but is very limited in many other Chinese provinces including Guangdong province.

Clonorchiasis is the most serious and important parasitic zoonosis in Guangdong province where approximately 5.5 million people were infected with *C. sinensis *[4,6,23], which accounts for approximately one-third of the total population infected with *C. sinensis *in China [4,6]. The present investigation represents the first comprehensive survey of *C. sinensis *infection in its reservoir hosts dogs and cats in Guangdong province. The average prevalence of *C. sinensis *infection in dogs and cats were 20.5% and 41.8%, respectively, which is a little lower than that in some other surveys in other endemic provinces such as Guangxi [15] (Table 1), but were still unacceptably high. These results demonstrated that *C. sinensis *infection in dogs and cats was common in Guangdong province and would play an important role in the transmission of *C. sinensis *between animals and humans.

The prevalence of *C. sinensis *infection in dogs and cats was different among different geographical localities in Guangdong province. The prevalence was relatively higher in some of the cities located in the Pearl River Delta region, such as 39.1% in dogs in Shenzhen, 66.7% and 73.2% in cats in Jiangmen and Guangzhou, respectively. The Pearl River Delta region is economically the most important area in Guangdong province, and there is a high prevalence of *C. sinensis *infection in freshwater fish and shrimps which are the second intermediate hosts for *C. sinensis *[4,24]. In the present study, dogs and cats in some cities which are located out of the Pearl River Delta region were also seriously infected with *C. sinensis*, for example 69.4% and 36.1% in dogs in Shaoguan and Yunfu, and 60% in cats in Yunfu, respectively, indicating that strategies and measures against clonorchiasis should also be used in these regions and cities.

**Figure 1:**
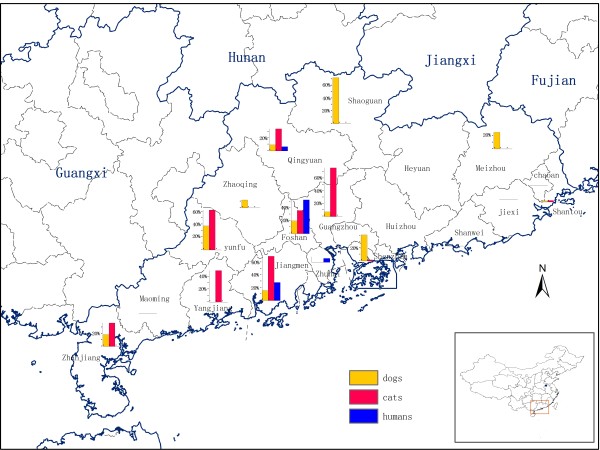
**Geographical locations and respective prevalence of *Clonorchis sinensis *infection in dogs, cats and humans **[23]**in Guangdong province**.

The prevalence of *C. sinensis *in dogs and cats was relatively lower in the seaside cities including Shantou, Zhanjiang, Maoming and Yangjiang (Tables 2 and 3, Figure 1). For example, the prevalence of *C. sinensis *in dogs (1.5%) and cats (2.1%) in Shantou (the eastern city of Guangdong province) was the lowest, which may due to differences in ecological, geographical and socioeconomic conditions. It is interesting to note that dogs in Shenzhen had a high prevalence of *C. sinensis *(39.1%), but the prevalence of *C. sinensis *in humans in this city was very low (1.6%, [23]). This is probably because Shenzhen is the biggest immigration city in southern China, the majority of its inhabitants were migrants from non-endemic areas, and they do not have the habit of eating raw or undercooked freshwater fish.

## Conclusions

The present survey demonstrated the high prevalence of *C. sinensis *infection in dogs and cats in southern China's subtropical Guangdong province where human clonorchiasis represents a major health problem, which poses a significant risk for public health. Integrated strategies and measures should be taken to tackle the problem of the high prevalence of *C. sinensis *infection in dogs and cats in this province, which, in turn, would have impact on the effective control and eventual elimination of human clonorchiasis.

## Competing interests

The authors declare that they have no competing interests.

## Authors' contributions

XQZ and XNZ conceived and designed the study, and critically revised the manuscript. SYH, JXC, MXC and HZ participated in study design, study implementation and manuscript revision. RQL, JDT, DHZ and HQS performed the experiments, analysed the data and drafted the manuscript. SYH, JXC, MXC and HZ helped in study implementation and data collection. All authors read and approved the final manuscript.
